# Supplementary material for: Artificial intelligence-based speech error detection to differentiate primary progressive aphasia variants
Source: Brain Commun. 2026 Sep 12;8(5):fcag353. doi: 10.1093/braincomms/fcag353 (PMC13613221; doi:10.1093/braincomms/fcag353)
Supplement: Supplementary file 1 [file fcag353_supplementary_data.zip › Supplementary Materials.pdf]

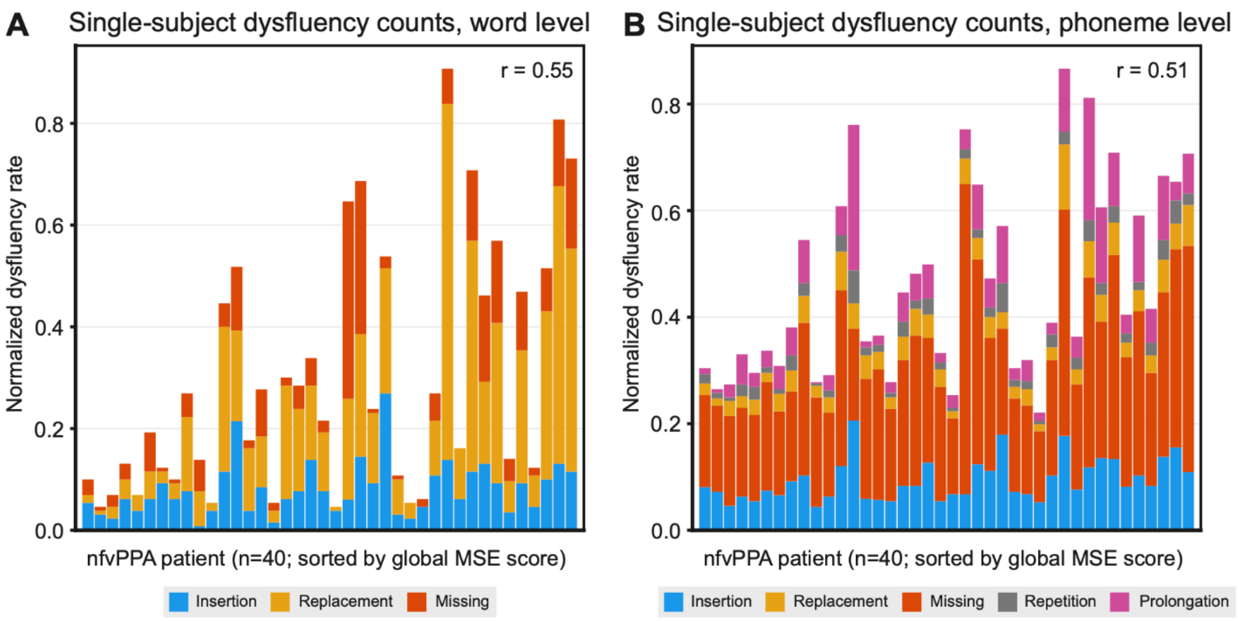

Supplementary Figure 1. Single-subject histograms of patients with nonfluent variant primary progressive aphasia (nfvPPA; n=40), sorted by ascending global motor speech evaluation (MSE) score, showing normalized lightweight Scalable Speech Dysfluency Modeling system (SSDM-L) dysfluency counts at the word (A) and phoneme (B) levels stacked by dysfluency type. The Pearson correlation coefficient *r* between overall dysfluency rate and global MSE score is displayed in the top right of each histogram
