# Supplementary material for: Artificial intelligence-based speech error detection to differentiate primary progressive aphasia variants
Source: Brain Commun. 2026 Sep 12;8(5):fcag353. doi: 10.1093/braincomms/fcag353 (PMC13613221; doi:10.1093/braincomms/fcag353)
Supplement: Supplementary file 1 [file fcag353_supplementary_data.zip › VideoSupplement_caption.docx]

**Supplemental Video S1. Video demonstration of the lightweight Scalable Speech Dysfluency Modeling system’s pipeline in a participant with logopenic primary progressive aphasia reading the Grandfather passage.**

Bottom left: Scrolling audio waveform represents the input to the lightweight Scalable Speech Dysfluency Modeling system’s pipeline. Playhead indicated with vertical red line. Table above shows word-level (top) and phoneme-level (bottom) transcripts for currently spoken word as well as automatically classified dysfluency type.

Right: Ticker feed of recent and upcoming words; current word (i.e., table contents) is highlighted in yellow.

Top left: Updating histograms of word-level (top) and phoneme-level (bottom) dysfluency counts.

**Supplemental Video S2. Video demonstration of the lightweight Scalable Speech Dysfluency Modeling system’s pipeline in a participant with non-fluent primary progressive aphasia reading the Grandfather passage.**

Bottom left: Scrolling audio waveform represents the input to the lightweight Scalable Speech Dysfluency Modeling system’s pipeline. Playhead indicated with vertical red line. Table above shows word-level (top) and phoneme-level (bottom) transcripts for currently spoken word as well as automatically classified dysfluency type.

Right: Ticker feed of recent and upcoming words; current word (i.e., table contents) is highlighted in yellow.

Top left: Updating histograms of word-level (top) and phoneme-level (bottom) dysfluency counts.
